# Clinical and Hematological Manifestation of Dengue Patients in 2022 Outbreak: A Tertiary Care Hospital‐Based Cross‐Sectional Study

**DOI:** 10.1002/hsr2.70356

**Published:** 2025-01-17

**Authors:** Md. Jubayer Hossain, Manisha Das, Muhibullah Shahjahan, Md. Wahidul Islam, Syeda Tasneem Towhid

**Affiliations:** ^1^ Population Health Studies Division Center for Health Innovation, Research, Action, and Learning–Bangladesh (CHIRAL Bangladesh) Dhaka Bangladesh; ^2^ Dhaka Medical College and Hospital Dhaka Bangladesh; ^3^ Department of Microbiology Jagannath University Dhaka Bangladesh

**Keywords:** Bangladesh, clinical manifestations, dengue, dengue outbreak, hematological manifestations

## Abstract

**Background and Aims:**

Dengue is a mosquito‐borne viral disease that frequently causes seasonal outbreaks in Bangladesh, particularly during the monsoon months from June to September. Recent outbreaks have shown significant shifts in clinical manifestations, including changes in the timeframe and serotype mixing. This study focused on the clinical and hematological profiles of patients during the 2022 outbreak, which was notably severe.

**Methods:**

This quantitative cross‐sectional study at Dhaka Medical College and Hospital classified 67 dengue‐positive patients as having dengue fever (DF), dengue fever with warning signs (DFWS), and severe dengue (SD). Fisher's exact test, Pearson's chi‐squared test, and Wilcoxon rank‐sum test were conducted for statistical analysis using the R software (version 4.2), with significance established at *p* < 0.05.

**Results:**

Predominantly male patients (76%) aged 21−40 (63%) and middle‐class patients from Dhaka (57%) formed the majority. Most patients (45%) lacked a regular drainage system. Laboratory tests showed 63% NS1 antigen (nonspecific antigen 1) positivity, and 16% tested positive for both NS1 and IgM (Immunoglobulin M). Symptoms included headache (84%), retroorbital pain (51%), joint pain (75%), myalgia (72%), vomiting (75%), abdominal pain (69%), diarrhea (39%), and shock (48%). Thrombocytopenia was present in 61% of patients, with 79% showing elevated hematocrit (HCT) levels. Pedal edema, pleural effusion, and ascites were observed in DF and DFWS cases, while SD cases exhibited distinct HCT level variations.

**Conclusion:**

Accurate clinical and laboratory assessments are vital for the treatment and prediction of dengue outcomes in Bangladesh, given the severe manifestations of dengue shock syndrome (DSS). It is characterized by headache, muscle pain, thrombocytopenia, anemia, leukopenia, and potential neurological complications or mortality. Severe cases involve critical plasma leakage, leading to DSS with fluid accumulation, respiratory distress, and potential multiorgan involvement. Enhancing physician awareness regarding dengue case detection and management, along with routine dengue testing, are recommended for early diagnosis and improved patient outcomes.

**List of Abbreviations**
BDT: Bangladeshi taka
DF: dengue fever
DFWS: dengue fever with/without warning sign
DSS: dengue shock syndrome
ELISA: enzyme‐linked immunosorbent assay
GIT: gastrointestinal tract
HCT: hematocrit
NS1: nonspecific Ag I
WBC: white blood cell

## 1 Introduction

Dengue fever (DF) is an arthropod‐borne viral disease caused by the dengue virus (DENV), a single‐stranded RNA virus of the genus Flavivirus, which is transmitted to humans by infected mosquitoes of the species *Aedes aegypti* and occasionally *Aedes albopictus* [1]. Dengue can cause various clinical symptoms, from typical influenza symptoms to severe bleeding or plasma leakage, resulting in dengue shock [2]. The main clinical features of severe dengue (SD) are hemorrhage and hypovolemic shock caused by increased vascular permeability and plasma leakage [3]. Patients typically present with fever, arthralgia, myalgia, retroorbital pain, rash, subconjunctival hemorrhage, respiratory symptoms, gastrointestinal (GI) disorders, low platelet count, and abnormal liver function test results [4]. Clinical manifestations of dengue infection are usually suspected and confirmed by hematological and microbiological laboratory tests such as viral antigen detection, specific serology, or virus isolation in cell cultures [5]. Studies have shown that the NS1 protein a highly conserved nonstructural glycoprotein (46 kDa) encoded by most flaviviruses present early in DENV infection. In the infected cell, it is formed as a soluble monomer. It dimerizes after posttranslational modification in the lumen of the endoplasmic reticulum and NS1 antigen capture enzyme‐linked immunosorbent assays (ELISA) can be used to diagnose DENV infection [6]. ELISA may be defined as highly sensitive chemical analysis, has been the mainstay of detection of various types of proteins, including cytokines, lymphokines, and phosphoproteins [7]. Of all the nations under observation since 1980, 17 were from the Americas, where Brazil, Peru, and Mexico had the highest reported caseloads. This region is responsible for most of the global burden. During the summer, autochthonous dengue cases have been documented in France, Italy, and Spain, despite the fact that dengue is not endemic to Europe [8]. In 11 African countries, the number of cases has increased significantly [9]. DF is endemic to WHO regions of Africa, America, Eastern Mediterranean, Southeast Asia, Western Pacific, and the Caribbean [10, 11, 12]. DF is an endemic disease in over 100 countries. DF affects approximately 2.5 billion people worldwide, with approximately 100 million new cases reported annually [13].

Millions of people in Southeast Asia, sub‐Saharan Africa, and Latin America are at risk of dengue infection [11, 12, 14, 15]. Bangladesh is geographically located in Southeast Asia, with a tropical monsoon and heavy rainfall from June to September. The Directorate General of Health Services (DGHS) reported a total of 100,201 confirmed dengue cases between January and December 2019, with 51,179 cases reported in Dhaka and the remaining 49,022 cases distributed across the country. This outbreak has posed significant health challenges to the city. As of November 20, 2022, Bangladesh has experienced a surge in dengue cases, including in Dhaka. From January 2022, the Ministry of Health and Family Welfare confirmed 52,807 laboratory‐confirmed dengue cases with 230 associated deaths, resulting in a case fatality rate (CFR) of 0.44% [16]. A devastating dengue epidemic was first reported in 2000. Between 2000 and 2017, 40,476 cases were reported during dengue outbreaks, most of which occurred during the monsoon season (May to August). It was discovered in all major cities, particularly in Dhaka [17]. The most common manifestations in the 2000 and 2002 outbreaks were high‐grade fever (100%) with a typical purpuric rash (55%), severe body aches (85%), and thrombocytopenia (56.7%) [18]. In 2008 and 2010, two observational studies in our study place concluded that fever, GI symptoms, and bleeding manifestations with normal platelet counts were more common and were associated with increased fatalities [19, 20]. The worst outbreak in Bangladesh has ever experienced occurred in 2019, beginning in the city of Dhaka and spreading to the entire nation [21]. All cases of suspected and actual dengue had fevers. Other frequent symptoms include retroorbital pain, nausea/vomiting, severe headaches, and abdominal pain [22]. Atypical symptoms are also becoming more prevalent with the disease burden but are frequently overlooked due to ignorance. Recently, dengue has become endemic in Bangladesh; however, a surge in dengue cases has begun in June 2022 [15, 23, 24]. All eight divisions in the country have reported cases and deaths. Between January 1 and November 20, 2022, 52,807 dengue cases and 230 related deaths (CFR = 0.44%) were reported [25]. This was the second largest outbreak since 2000, with the largest outbreak occurring in 2019. The national guideline in dengue control and prevention is methodologically followed in almost every hospital after categorizing the case by overall assessment, diagnosis phase, and severity, followed by medical management [26]. The current dengue outbreak is unusual in scale and seasonality, including the pattern of symptoms and duration of DF types. This may be due to DENV infection alteration with different serotypes compared with previous years [1]. Multiple circulating DENV serotypes in Bangladesh may exacerbate the disease severity through serial infections. Bangladesh, like many regions, has experienced a dynamic landscape of DENV serotypes, with initial dominance of DEN‐3 in 2002 followed by a shift toward DEN‐1 and DEN‐2 from 2013 to 2016 [1, 15, 23]. Notably, recent epidemics have witnessed a resurgence in DEN‐3. This temporal variability in serotype circulation, coupled with the potential for repeated infections across serotypes, poses a significant public health concern because of the risk of increased disease severity in subsequent infections. This dynamic interplay between serotype diversity and reinfection warrants further investigation of its potential impact on the clinical manifestations of dengue infections in Bangladesh [1, 15]. Previous studies have shown a significant correlation between the shifting patterns of the clinical and hematological profiles in patients with dengue infection [27, 28]. Thus, this study aimed to investigate the shifting patterns of clinical and hematological profiles in patients presenting with dengue infections and to identify a strategy to lessen the burden of the epidemic in Bangladesh during the rainy season.

## 2 Methods

### 2.1 Study Location and Participants

The study was conducted at Dhaka Medical College and Hospital (DMCH), which is the oldest tertiary‐level hospital in the heart of Dhaka. Currently, it has 2600 beds (including 300 beds in the Burn and Plastic Surgery Unit). The hospital offers all major medical and surgical specialties, as well as subspecialties. Almost all of the modern diagnostic facilities are available. With 234 doctors, 140 interns, 560 nurses, and 1100 other staff members spread across 34 departments and 42 wards, DMCH is dedicated to providing 24 h healthcare services to both inpatients and outpatients, catering to approximately 2 million inhabitants of Bangladesh. Dhaka, the capital city and a dengue hotspot, has recently witnessed multiple dengue outbreaks [29]. It is essential to acknowledge that this outbreak is ongoing beyond November 2022, and further updates may have emerged. Suspected dengue cases admitted in November 2022 were included in the study, and the analysis was limited to confirmed patients who met the inclusion criteria.

### 2.2 Study Design

This retrospective cross‐sectional investigation was conducted on individuals who received a serological diagnosis of DENV infection determined by the presence of NSI or IgM antibodies. Comprehensive blood count assessments were performed for all hospitalized patients during their admission.

### 2.3 Inclusion Criteria

Participants involved in this study who fulfilled the requirements (i) admitted to DMCH, (ii) patients provided consent to participate in the study from the dengue ward or ICU of the hospital, (iii) male and female patients aged > 14 years, and (iv) patients were diagnosed serologically based on positive NS1 antigen or IgM antibody test results.

### 2.4 Exclusion Criteria

Exclusion criteria include (i) patients with other viral or bacterial infections after routine laboratory testing, (ii) not provided consent, and (iii) patients whose clinicians consider them too unwell to participate.

### 2.5 Data Collection Tools

Data was collected using an Open Data Kit (ODK Software, KoboCollect). This study collected the patients' clinical information using a structured questionnaire. The research team developed a questionnaire based on an extensive literature review [30, 31, 32, 33]. Then, it was validated by a clinical physician to check the appropriateness and specificity of DF‐related questions. The questionnaire included detailed information about the study, participant consent, demographic information, and clinical profiles including abdominal pain, hepatomegaly (enlargement of liver size), diarrhea (passing loose stool), headache, retroorbital pain (pain behind the head), platelet profiles, and hematological profiles.

### 2.6 Data Collection

Face‐to‐face interviews were conducted to collect data from the participants. Written informed consent was obtained from all respondents, but their parents requested their consent from the minor ones. The following information was obtained at the time of data collection: demographic variables included socioeconomic (age, sex, blood group, employment status, transfusion status, economic class‐ middle and lower [34, 35], total cost of hospital stay, years resident in Dhaka) and environmental (use of mosquito net, regular cleaning of drainage system, regular visits to doctors). The questionnaire included detailed information about the study, participant consent, demographic information, and clinical profiles. Abdominal pain, hepatomegaly (enlargement of liver size), diarrhea (passing loose stool), headache, retroorbital pain (pain behind the head), platelet profiles, and hematological profiles. Clinical variables included medical history (hypertension, diabetes, asthma, allergy) and acute illness (laboratory diagnosis of dengue test, admission status, and length of hospitalization). Hematological variables included complete blood count (minimum and maximum WBC count, total leukocyte count [TLC], hematocrit [HCT], and platelet count), physical examination findings (pedal edema, ascites, pleural effusion), outcome (death or discharge), and laboratory tests (e.g., NS1 antigen, IgM antibody, and ELISA). Serum NS1 levels are closely linked with DENV viremia; they are evident at the start of the febrile illness and peak 3−5 days after the onset of symptoms [36]. The serological assays have several benefits since they are based on the identification of IgM and IgG antibodies against the envelope protein. They don't require expensive equipment and are reasonably priced [37]. Molecular methods are established on DENV RNA detection by real‐time RT‐PCR (reverse transcriptase‐polymerase chain reaction) [38].

### 2.7 Statistical Analysis

Patients in this study were classified into three categories based on their clinical symptoms: DF without warning signals (DF), DF with warning signs (DFWS), and SD. For better clinical management, the 2009 WHO classification for dengue was developed to make it simple for doctors to classify patients based on their clinical presentations. This new classification, which replaces the 1997 categorization of dengue into undifferentiated fever, DF, and dengue hemorrhagic fever, aims to provide more clarity on the severity of clinical presentations [39]. These divisions were formed using clinical criteria that provide a systematic method for classifying individuals' DF severity [40]. Clinicians may independently consider clinical and biological criteria related to hemorrhagic tendencies and plasma leakage as warning signs and thus adapt appropriate patient management at given times during hospitalization. Moreover, besides dengue shock syndrome, the WHO 2009 classification proposes definitions for non‐DHF types of severe DF with severe organ involvement. In assessing the association between categorical variables, Fisher's exact test was used in instances of smaller sample sizes or when the expected frequencies in contingency tables were too low for the reliable application of Pearson's chi‐squared test. Pearson's chi‐squared test was employed for larger samples where its assumptions were met. For numeric variables, the Wilcoxon rank‐sum test was used to compare median values across the DF classifications (DF, DFWS, SD), particularly where the data did not meet the normality assumptions required for parametric tests. All statistical analyses were performed using R software (version 4.2). A significance level of *p* < 0.05 was used for all tests to determine the presence of statistically significant differences or associations.

## 3 Results

The study participants comprised 76% (*n* = 51) male and 24% (*n* = 16) female patients. Most patients (63%, *n* = 42) were aged between 21 and 40 years. The blood group distribution was O+ (28%, *n* = 28), B+ (24%, *n* = 16), A+ (15%), AB+ (9%, *n* = 9), and B− (3%, *n* = 3), and 21% (*n* = 21) were unsure of their blood type. Of the patients, 58% (*n* = 39) were employed, and 42% (*n* = 28) were unemployed. Twenty‐four percent (*n* = 16) of the patients received a blood transfusion during their hospital stay. The economic class was primarily the middle class, 82% (*n* = 55) with 18% (*n* = 15) classified as the lower class according to the recent literature [34, 41]. Seventy‐five percent (*n* = 50) of the patients had a hospital stay that cost more than 3000 BDT (Bangladeshi taka, 26$). The number of years of residency in Dhaka varied, with 39% (*n* = 26) of the patients having been residents for 10 or more years. A mosquito net was used in 70% (*n* = 47) of the patients, while 30% (*n* = 20) did not. Regular cleaning of drainage systems was reported by 45% (*n* = 30) of the patients, and 70% (*n* = 47) did not regularly visit a doctor (Table 1).

**Table 1:** Sociodemographic characteristics.

Characteristic	*N* = 67
Patient's age	
15–20	10 (15%)
21–40	42 (63%)
41–60	15 (22%)
Gender	
Male	51 (76%)
Female	16 (24%)
Blood group	
O+	19 (28%)
B+	16 (24%)
Don't know	14 (21%)
A+	10 (15%)
AB+	6 (9.0%)
B−	2 (3.0%)
Employment status	
Employed	39 (58%)
Unemployed	28 (42%)
Transfusion status	
No	51 (76%)
Yes	16 (24%)
Economic class	
Middle	55 (82%)
Lower	12 (18%)
The total cost of the stay in the hospital in Taka	
3000 or more	50 (75%)
< 3000	15 (22%)
Don't know	2 (3.0%)
Years resident in Dhaka	
Nonresident	29 (43%)
10+ years	26 (39%)
≤ 10 years	12 (18%)
Use of mosquito net	
Yes	47 (70%)
No	20 (30%)
Regular cleaning of the drainage system	
No	37 (55%)
Yes	30 (45%)
Regular visits to doctors	
No	47 (70%)
Yes	20 (30%)

Of the clinical characteristics of 67 admitted patients, 96% (*n* = 64) had no history of hypertension, and only 4.5% (*n* = 3) had hypertension. Similarly, 94% (*n* = 63) of the patients had no history of diabetes and 6% (*n* = 4) had diabetes. The majority of patients (99%, *n* = 66) had no history of asthma, and 90% (*n* = 60) had no other comorbidities. Thirty (*n* = 20%) of patients had a history of allergies, whereas 57% had no comorbidities. Most patients (63%, *n* = 42) were diagnosed based on a positive NS1 antigen test, whereas 16% (*n* = 11) were positive for both NS1 and IgM. A small percentage (4.5%, *n* = 3) had only IgM antibody positivity and 16% (*n* = 11) had no laboratory diagnosis. Almost all patients (97%, *n* = 65) were admitted to the ward, while only 3% (*n* = 2) required ICU admission, 30% (*n* = 20) stayed for 7 or more days, and 70% (*n* = 47) were discharged in less than 7 days (Table 2).

**Table 2:** Clinical characteristics of admitted cases.

Characteristic	*N* = 67
Hypertension	
No	64 (96%)
Yes	3 (4.5%)
Diabetes	
No	63 (94%)
Yes	4 (6.0%)
Asthma	
No	66 (99%)
Yes	1 (1.5%)
Others^a^	
No	60 (90%)
Yes	7 (10%)
Allergy	
No	47 (70%)
Yes	20 (30%)
Lab diagnosis	
NS1 antigen positive	42 (63%)
Both NS1 and IgM positive	11 (16%)
None	11 (16%)
IgM antibody positive	3 (4.5%)
Admission	
Ward	65 (97%)
ICU	2 (3.0%)
Length of hospitalization	
Below 7 days	47 (70%)
7 or more days	20 (30%)

a Cardiac problems, renal disease, generalized weakness, and so forth.

Among the 67 patients, 84% (*n* = 56) presented with headaches, and 51% (*n* = 34) experienced retroorbital pain. A rash was present in 34% (*n* = 23) of the patients, while 75% (*n* = 50%) and 72% (*n* = 48) experienced joint pain and myalgia, respectively. Vomiting was reported in 75% (*n* = 50) of cases, whereas nasal or gum bleeding was observed in only 19% (*n* = 13). Hematemesis was reported in 13% (*n* = 9) of the cases. Abdominal pain was present in 69% (*n* = 46) of the cases, while diarrhea was present in 39% (*n* = 26). Hepatomegaly was present in 19% (*n* = 13) of the cases, while shock was present in 48% (*n* = 32) (Table 3).

**Table 3:** Presenting symptoms among admitted patients.

Characteristic	*N* = 67
Headache	
Present	56 (84%)
Absent	11 (16%)
Retro orbital pain	
Present	34 (51%)
Absent	33 (49%)
Rash	
Absent	44 (66%)
Present	23 (34%)
Joint pain	
Present	50 (75%)
Absent	17 (25%)
Myalgia	
Present	48 (72%)
Absent	19 (28%)
Vomiting	
Present	50 (75%)
Absent	17 (25%)
Nasal or gum bleeding	
Absent	54 (81%)
Present	13 (19%)
Hematemesis	
Absent	58 (87%)
Present	9 (13%)
Abdominal pain	
Present	46 (69%)
Absent	21 (31%)
Diarrhea	
Absent	41 (61%)
Present	26 (39%)
Hepatomegaly	
Absent	54 (81%)
Present	13 (19%)
Shock	
Absent	35 (52%)
Present	32 (48%)

The patients in this study had a wide range of white blood cell counts, with a minimum count of 33,796 cu.mm and a maximum count of 106,728 cu.mm. Most patients (78%) had a TLC of 4000−11,000 cu.mm. Additionally, a significant number of patients (18%) had a TLC of > 11,000 cu.mm. Regarding HCT or packed cell volume (PCV) percentage, most patients (79%) had values greater than 20%, and 21% had values equal to or less than 20%. Regarding platelet count, nearly half of the patients (49%) had platelet counts less than 100,000 cu.mm. Only 7.5% of the patients had platelet counts greater than 300,000 cu.mm, while 43% had counts within the 100,000−300,000 cu.mm range. Regarding clinical signs, 15% and 25% of patients had pedal edema and ascites, respectively. Moreover, 24% of the patients had pleural effusion and 54% had DF (Table 4).

**Table 4:** Hematological parameters of dengue patients.

Characteristic	*N* = 67
Patient's minimum WBC count	33,796 (123,473)
Patient's maximum WBC count	106,728 (502,171)
Total leukocyte count (TLC)	
4000−11,000 cu.mm	52 (78%)
> 11,000 cu.mm	12 (18%)
< 4000 cu.mm	3 (4.5%)
Hematocrit (HCT) or PCV (%)	
> 20	53 (79%)
≤ 20	14 (21%)
Platelet count	
< 100,000 cu.mm	33 (49%)
100,000−300,000 cu.mm	29 (43%)
> 300,000 cu.mm	5 (7.5%)
Pedal edema	
No	57 (85%)
Yes	10 (15%)
Ascites	
No	50 (75%)
Yes	17 (25%)
Pleural effusion	
No	51 (76%)
Yes	16 (24%)
DF	
Yes	36 (54%)
No	31 (46%)

*Note:* Mean (SD); *n* (%).

A comparative analysis was conducted between patients with and without warning signs of DF to identify potential associations with the demographic and clinical parameters. The results presented in Table 5 reveal that sex, pedal edema, ascites, and pleural effusion were significantly associated with DF (*p* < 0.05), as indicated by the Chi‐square test. However, age and hematological parameters did not demonstrate any statistically significant association (*p* > 0.05).

**Table 5:** Comparison of parameters among those dengue fever.

Characteristic	Overall (*N* = 67)	No (*N* = 31)	Yes (*N* = 36)	*p* value
Patient's age				0.11
15–20	10 (15%)	2 (6.5%)	8 (22%)	
21–40	42 (63%)	23 (74%)	19 (53%)	
41–60	15 (22%)	6 (19%)	9 (25%)	
Gender				**0.039**
Male	51 (76%)	20 (65%)	31 (86%)	
Female	16 (24%)	11 (35%)	5 (14%)	
Patient's minimum WBC count (cu.mm)	33,796 (123,473)	12,117 (12,503)	52,464 (166,854)	0.3
Patient's maximum WBC count (cu.mm)	106,728 (502,171)	34,147 (46,271)	169,228 (681,918)	0.8
Total leukocyte count (TLC)				> 0.9
4000−11,000 cu.mm	52 (78%)	25 (81%)	27 (75%)	
> 11,000 cu.mm	12 (18%)	5 (16%)	7 (19%)	
< 4000 cu.mm	3 (4.5%)	1 (3.2%)	2 (5.6%)	
Hematocrit (HCT) or PCV (%)				0.4
> 20	53 (79%)	23 (74%)	30 (83%)	
≤ 20	14 (21%)	8 (26%)	6 (17%)	
Platelet count				0.4
< 100,000 cu.mm	33 (49%)	18 (58%)	15 (42%)	
100,000−300,000 cu.mm	29 (43%)	11 (35%)	18 (50%)	
> 300,000 cu.mm	5 (7.5%)	2 (6.5%)	3 (8.3%)	
Pedal edema				**< 0.001**
No	57 (85%)	21 (68%)	36 (100%)	
Yes	10 (15%)	10 (32%)	0 (0%)	
Ascites				**< 0.001**
No	50 (75%)	14 (45%)	36 (100%)	
Yes	17 (25%)	17 (55%)	0 (0%)	
Pleural effusion				**< 0.001**
No	51 (76%)	15 (48%)	36 (100%)	
Yes	16 (24%)	16 (52%)	0 (0%)	

*Note:* Bold values indicate statistically significant.

In the present study, patients with and without warning signs were compared; the results are presented in Table 5. Our findings demonstrate that several clinical factors, including sex, pedal edema, ascites, and pleural effusion, were significantly associated with DF with warning signs (*p *< 0.05). Table 6 compares various parameters among patients diagnosed with DF with warning signs.

**Table 6:** Comparison of parameters among those with dengue fever with or without warning signs (DFWS).

Characteristic	Overall (*N* = 67)	No (*N* = 36)	Yes (*N* = 31)	*p* value
Patient's age				0.11
15–20	10 (15%)	8 (22%)	2 (6.5%)	
21–40	42 (63%)	19 (53%)	23 (74%)	
41–60	15 (22%)	9 (25%)	6 (19%)	
Gender				**0.039**
Male	51 (76%)	31 (86%)	20 (65%)	
Female	16 (24%)	5 (14%)	11 (35%)	
Patient's minimum WBC count (cu.mm)	33,796 (123,473)	52,464 (166,854)	12,117 (12,503)	0.3
Patient's maximum WBC count (cu.mm)	106,728 (502,171)	169,228 (681,918)	34,147 (46,271)	0.8
Total leukocyte count (TLC)				> 0.9
4000−11,000 cu.mm	52 (78%)	27 (75%)	25 (81%)	
> 11,000 cu.mm	12 (18%)	7 (19%)	5 (16%)	
< 4000 cu.mm	3 (4.5%)	2 (5.6%)	1 (3.2%)	
Hematocrit (HCT) or PCV (%)				0.4
> 20	53 (79%)	30 (83%)	23 (74%)	
≤ 20	14 (21%)	6 (17%)	8 (26%)	
Platelet count				0.4
< 100,000 cu.mm	33 (49%)	15 (42%)	18 (58%)	
100,000−300,000 cu.mm	29 (43%)	18 (50%)	11 (35%)	
> 300,000 cu.mm	5 (7.5%)	3 (8.3%)	2 (6.5%)	
Pedal edema				**< 0.001**
No	57 (85%)	36 (100%)	21 (68%)	
Yes	10 (15%)	0 (0%)	10 (32%)	
Ascites				**< 0.001**
No	50 (75%)	36 (100%)	14 (45%)	
Yes	17 (25%)	0 (0%)	17 (55%)	
Pleural effusion				**< 0.001**
No	51 (76%)	36 (100%)	15 (48%)	
Yes	16 (24%)	0 (0%)	16 (52%)	

*Note:* Bold values indicate statistically significant.

Table 7 presents a comparison of various parameters among individuals with SD. The results indicated no statistically significant association between SD and the patient demographic parameters (*p* > 0.05). However, only hematological parameters HCT or PCV (%) indicated a significant association (*p* < 0.05).

**Table 7:** Comparison of parameters among that severe dengue.

Characteristic	Overall (*N* = 67)	No (*N* = 35)	Yes (*N* = 32)	*p* value
Patient's age				0.3
15–20	10 (15%)	4 (11%)	6 (19%)	
21–40	42 (63%)	25 (71%)	17 (53%)	
41–60	15 (22%)	6 (17%)	9 (28%)	
Gender				0.4
Male	51 (76%)	28 (80%)	23 (72%)	
Female	16 (24%)	7 (20%)	9 (28%)	
Patient's minimum WBC count (cu.mm)	33,796 (123,473)	27,226 (83,252)	40,982 (157,337)	0.5
Patient's Maximum WBC count (cu.mm)	106,728 (502,171)	55,191 (105,830)	163,097 (719,950)	0.5
Total leukocyte count (TLC)				0.4
4000−11,000 cu.mm	52 (78%)	25 (71%)	27 (84%)	
> 11,000 cu.mm	12 (18%)	8 (23%)	4 (13%)	
< 4000 cu.mm	3 (4.5%)	2 (5.7%)	1 (3.1%)	
Hematocrit (HCT) or PCV (%)				**0.046**
> 20	53 (79%)	31 (89%)	22 (69%)	
≤ 20	14 (21%)	4 (11%)	10 (31%)	
Platelet count				0.6
< 100,000 cu.mm	33 (49%)	15 (43%)	18 (56%)	
100,000−300,000 cu.mm	29 (43%)	17 (49%)	12 (38%)	
> 300,000 cu.mm	5 (7.5%)	3 (8.6%)	2 (6.3%)	
Pedal edema				0.3
No	57 (85%)	28 (80%)	29 (91%)	
Yes	10 (15%)	7 (20%)	3 (9.4%)	
Ascites				> 0.9
No	50 (75%)	26 (74%)	24 (75%)	
Yes	17 (25%)	9 (26%)	8 (25%)	
Pleural effusion				0.3
No	51 (76%)	25 (71%)	26 (81%)	
Yes	16 (24%)	10 (29%)	6 (19%)	

*Note:* Bold values indicate statistically significant.

## 4 Discussion

The key findings of this study underscore the substantial clinical relevance of various factors associated with the manifestation of DF presenting with warning signs, as indicated by the statistical significance levels (*p* < 0.05). Among these factors are sex, pedal edema, ascites, pleural effusion, and HCT or PCV. A noteworthy observation pertains to leukocyte count, with 71% of patients exhibiting counts falling within the range of 4000−11,000 cu.mm. HCT or PCV analysis revealed that a significant majority (79%) of patients demonstrated values exceeding 20%, whereas 21% exhibited values at or below this threshold. A particularly salient finding is the incidence of thrombocytopenia, with nearly half of the patients (49%) presenting with platelet counts < 100,000 cu.mm. These findings provide valuable insights into the clinical profile of DF, shedding light on factors that may serve as crucial indicators, thereby facilitating enhanced understanding and management of the disease.

The present study constitutes the first retrospective investigation undertaken in Bangladesh to analyze the clinical and hematological presentations of patients with dengue. While research concerning dengue outbreaks in Bangladesh exists, this work breaks new ground by focusing specifically on patient manifestations [15, 23, 24]. Additionally, results from lab data analysis allowed us to better understand the characteristics and clinical presentation of DF with warning signs.

Data on the dengue outbreak in 2022 showed that most patients are admitted to words to recover from DF [42]. The higher likelihood of males being affected by dengue than females, along with the majority representation of the 21−40 age group (63%) of dengue cases in 2022 [23, 43, 44, 45, 46, 47], can be attributed to factors such as differences in behavior and occupational exposure. Males may engage in activities that increase their exposure to dengue‐carrying mosquitoes, whereas certain occupations prevalent among the 21−40 age group may contribute to their higher risk. Biological factors and reporting biases may also play a role, with females potentially having a more robust immune response or differing healthcare‐seeking behaviors. However, further investigation is necessary to validate these explanations and to understand the specific context of the dengue outbreak. Only 24% of the patients received a blood transfusion after admission, while a study in South Kerala found the percentage to be 16.4% [43]. Dengue hemorrhagic fever or SD is a vital indication for blood transfusions. A report of fewer than one‐fourth of transfusions may indicate milder dengue complexities requiring proper ward treatment and monitoring [43].

In this study, the most frequent clinical presentations were headache, retroorbital pain, joint pain, myalgia (muscle pain), vomiting, and abdominal pain, consistent with other studies conducted in India, Pakistan, Saudi Arabia, and Taiwan [43, 44, 47, 48, 49]. The rash was observed in 34% and hepatomegaly in 21% of the cases in our study, whereas similar studies in Pakistan and Malaysia reported rashes in 56.3% and 26% of patients, respectively [45, 48]. Retroorbital pain is one of the most common features of DF. Although the specific pathophysiological mechanisms underlying dengue ocular problems remain uncertain, numerous investigations have suggested that an immune‐mediated process is a possible culprit [50, 51, 52]. In our study, 50% of patients complained of this condition, as observed in a study in Ethiopia in 2017 [27]. Studies in India have reported a low incidence (10%) of retroorbital pain [43, 48]. Nasal or gum bleeding, one of the symptoms of dengue hemorrhagic fever, was reported in 19% of patients, whereas a study in Saudi Arabia found only 5.6% in OPD settings in 2019 [49]. A frequent clinical manifestation of dengue is bleeding diathesis, which is caused by blood vessel leakage and low platelet count. This results from the interaction of DENV with host cells, which releases too many cytokines and activates the immune system, altering the vascular endothelium and producing mononuclear cell infiltration and perivascular edema [27]. Organ involvement, hepatomegaly, and ascites were clinically diagnosed in only 21% and 24% of cases, respectively, whereas only 4.7% and 6.3% of cases were reported in India during the 2017 outbreak [43]. The variations in the clinical presentation in different studies might be due to differences in the strain of the virus and its virulence factors.

Hematological parameters of DF, including platelet, leukocyte, and HCT levels, are commonly assessed. A rational outcome would typically be decreased platelet and leukocyte levels and increased HCT levels by more than 20%. Under dengue conditions, leukopenia started on Day 2 and peaked on Day 5. Bone marrow examination revealed mild hypocellularity during the first 7 days of fever and normal cellularity during the convalescent phase; one theory regarding the occurrence of leukopenia in dengue infection cases was caused by the destruction or inhibition of myeloid progenitor cells [28]. In this study, the most significant finding regarding TLC was that 71% of patients fell within the normal range. Only a small percentage (5.9%) exhibited leukopenia, which was significantly lower than that reported in a previous study (33.8%) [49]. Similar findings have been reported by Sri Lankan [53]. Thrombocytopenia, characterized by low platelet counts, was present in nearly half of the population (49%), aligning with findings from studies conducted in India, Sri Lanka, Pakistan, and Nepal [42, 46, 53, 54]. The development of thrombocytopenia in dengue infection could be attributed to factors such as the inhibition of megakaryopoiesis and promotion of apoptotic cell death in a subpopulation of early megakaryocytic progenitors, as well as the direct interaction and activation of platelets by the DENV [55]. Furthermore, an elevated HCT level (> 20%) was observed in 79% of the patients, which shows some variation compared to previous studies. This discrepancy may be due to the use of different cut‐off values in previous studies.

The present study identified several clinical parameters that were strongly associated with DF with warning signals. These variables included pleural effusion, ascites, pedal edema, and gender [32, 56, 57, 58]. Based on these significant relationships, these clinical characteristics may serve as major indicators or predictors of DF with warning signals. There is a need for more research into potential gender‐related aspects that affect the disease because gender variations may potentially affect how dengue symptoms emerge and the severity of the disease. Identifying these clinical characteristics and warning signs of DF has consequences for patient management, early detection, and risk assessment [23]. It allows medical professionals to identify patients who are more likely to experience SD and allows for focused monitoring and timely medical interventions. Validating these findings and examining the underlying processes by which these clinical parameters lead to the emergence of warning signals in DF requires additional study. Additionally, exploring the potential interactions between these variables and different demographic or laboratory characteristics may offer a complete insight into the course and severity of the condition.

## 5 Limitations

In our study, we controlled for various factors such as age, sex, and specific clinical symptoms that are known to influence the manifestation of DF. We recognize that there could be additional confounding factors, such as socioeconomic status or geographic variation, that were not directly accounted for in our analysis. This study had notable limitations, primarily stemming from the restricted sample size due to COVID‐19‐related constraints. Government‐imposed restrictions on movement, particularly in hospital areas, unless under critical conditions, significantly constrain access to potential participants. Additionally, the pandemic context led to acute patients' reluctance to stay in hospital settings, opting for home‐based treatment, thus further limiting the study's sample size. Additionally, the comprehensive evaluation of all participants and serotyping of dengue cases are hindered by insufficient resources and facility availability. Furthermore, the scarcity of complete information in existing studies has impeded in‐depth analysis encompassing a thorough comparison of various outbreaks.

## 6 Conclusions

This study investigated factors associated with DF presenting with warning signs. While leukocyte count fell within the normal range for most patients (71%), nearly half exhibited thrombocytopenia (platelet count < 100,000). Sex, fluid buildup (edema, ascites, pleural effusion), and HCT were statistically significant indicators for dengue with warning signs. The study highlighting headache, myalgia, thrombocytopenia, anemia, and leukopenia contributes valuable insights for clinicians diagnosing and managing DF. Understanding these characteristic clinical features and laboratory abnormalities is crucial for guiding therapy and determining prognosis in patients with suspected dengue infection.

## Author Contributions

**Md. Jubayer Hossain:** conceptualization, investigation, formal analysis and interpretation of data, writing–original draft, writing–review and editing, project administration. **Manisha Das:** data curation, investigation, writing–original draft, writing–review and editing. **Muhibullah Shahjahan:** data curation, writing–original draft, writing–review and editing. **Md. Wahidul Islam:** data curation, interpretation of data, writing–original draft, writing–review and editing. **Syeda Tasneem Towhid:** project administration and funding acquisition.

## Ethics Statement

This study was approved by the Ethical Review Committee of CHIRAL Bangladesh (Reference No: CHIBAN05NOV2022‐0007). Informed consent was obtained from all participants, and data analysis was conducted anonymously. Patient identification was performed using registration numbers (RNs) extracted from medical records. Before the data analysis, each case was assigned unique numerical codes to ensure confidentiality.

## Consent

The authors have nothing to report.

## Conflicts of Interest

The authors declare no conflicts of interest.

## Transparency Statement

The lead author Md. Jubayer Hossain affirms that this manuscript is an honest, accurate, and transparent account of the study being reported, that no important aspects of the study have been omitted, and that any discrepancies from the study as planned (and, if relevant, registered) have been explained.

## Data Availability

The data that support the findings of this study are openly available in Dengue_Clinical_Presentation at https://github.com/chiralbd/Dengue_Clinical_Presentation. The data sets used and/or analyzed during the current study are available from the corresponding author upon reasonable request.
